# The Role of Body Size and Shape in Understanding Competitive Interactions within a Community of Neotropical Dung Beetles

**DOI:** 10.1673/031.011.0113

**Published:** 2011-02-04

**Authors:** Malva I. M. Hernández, Leandro R. Monteiro, Mario E. Favila

**Affiliations:** ^1^ Departamento de Ecologia e Zoologia, Universidade Federal de Santa Catarina, Caixa Postal 476, Florianópolis, SC, Brazil 88010-970; ^2^ Department of Biological Sciences, The University of Hull, Hull, HU6 7RX, United Kingdom; ^3^ Instituto de Ecología A.C., Apartado Postal 63, Xalapa, 91000, Veracruz, Mexico

**Keywords:** Brazil, Coleoptera, geometric morphometrics, inter-specific competition, Scarabaeinae

## Abstract

Geometric morphometrics is helpful for understanding how body size and body shape influence the strength of inter-specific competitive interactions in a community. Dung beetles, characterized by their use of decomposing organic material, provide a useful model for understanding the structuring of ecological communities and the role of competition based on their size and morphology. The relationship between body size and shape in a dung beetle community from the Atlantic Forest in Serra do Japi, Brazil was analyzed for 39 species. Fifteen anatomical landmarks on three-dimensional Cartesian coordinates were used to describe both the shape and the size of the body of each species on the basis of the centroid located along homologous points in all of the species. The first vector of a principal components analysis explained 38.5% of the morphological variation among species, and represents a gradient of body shape from elongated, flattened bodies with narrow abdomen to rounded or convex bodies. The second component explained 17.8% of the remaining variation in body shape, which goes from species with an abdomen that is larger than the elytra to species with constricted abdomens and large elytra. The relationship between body size and shape was analyzed separately for diurnal and nocturnal species. In both guilds not only were there differences in body size, but also in body shape, suggesting a reduction in their level of competition.

## Introduction

Species morphology has always been considered an important factor that affects the patterns of inter- and intra-specific competition in ecological communities. Within a single community, species from the same taxonomic group that have a similar body size often exploit similar resources and are therefore likely to compete more strongly with each other than with species which are less similar (Warren and Lawton 1987; Juliano and Lawton 1990). According the principle of competitive exclusion two species cannot occupy the same ecological niche (Gause 1934). However, resource partitioning should reduce competition and allow for species coexistence (Begon et al. 1996; Tilman 2007). Animals with different body shapes supposedly exploit some resources more efficiently than other species do, and this reduces competition and promotes species coexistence (Hutchinson & MacArthur 1959). Body size and shape are often correlated with various other individual traits such as physiology, behavior, and metabolism (e.g. life expectancy, locomotion, and fecundity), as well as ecological characteristics such as population density, the distribution of relative species abundance, and competitive ability (Peters 1983; Calder 1984; Schmidt-Nielsen 1984; Morse *et al*. 1988; Lawton 1991; Blackburn and Gaston 1997). Although coexistence is often attributed to interspecific differences in morphology, direct evidence is relatively rare (Gurd 2007).

Developments in geometric morphometrics have been successful in combining the fields of geometry, biology, and statistics for the purpose of doing more reliable comparative studies (Bookstein 1982, 1991; Rohlf and Marcus 1993). Insects are ideal for this type of study, not only because of their hyperabundance, but also because they possess a well defined exoskeleton (see Adams and Funk 1997; Pretorius *et al*. 2000; Pretorius and Scholtz, 2001). Within the insects, beetles generally have an oval or elongated shape and a convex body. Their robust exoskeleton makes measuring them a straightforward task. Additionally, the complete metamorphosis that occurs in beetles reduces the difficulty presented by allometric growth in immature stages, as measurements are limited to adult beetles.

The species of the subfamily Scarabaeinae (Scarabaeidae), commonly known as dung beetles, are characterized by the use of decomposing organic material by adults and larvae as a food source. There are 25 to 70 species in tropical rain forests, but as many as 124 species in African savannas (Favila and Halffter 1997). Several etoecological differences have been invoked to explain the high diversity of dung beetle species in tropical ecosystems: food relocation system with burying, roller, and dweller species; diel activity including nocturnal, crepuscular, and diurnal species; food preference with coprophagous, copronecrophagous, and necrophagous species; and finally there are stenotopic and eurytopic species with different temporal activity patterns over an annual period (Halffter and Matthews 1966; Hanski and Koskela 1977; Halffter and Edmonds 1982; Giller and Doube 1989, 1994; Hanski and Cambefort 1991; Halffter *et al*. 1992; Davis 1996; Palestrini *et al*. 1998; Hernández 2002; Feer and Pincebourde 2005; Horgan and Fuentes 2005). The reduction in direct competition and the resulting coexistence of many species is expected to result from not only these etoecological differences, but also from morphometric variations. As such, dung beetles seem to be an ideal group for analyzing how morphology is related to niche segregation, resource partitioning, and the structuring of communities.

This paper presents an approach that uses geometric morphometrics to elucidate the competitive relationships in dung beetle assemblages in order to understand community structure and species coexistence. We predict that similar sized species will have different shapes where they overlap along some major resource continuum, and that sizeshape overlap will be low within nocturnal or diurnal species.

## Materials and Methods

Dung beetles were collected in an Atlantic Forest in the Serra do Japi, São Paulo, Brazil (23° 12′ to 23°22′ S and 46°53′ to 47°03′ W) at an altitude of 1000 MASL. Sampling was carried out between September 1997 and August 1998 at 6 different sites using 4 pitfall traps per site (a total of 24 pitfall traps) that were baited with human feces and left open for two days every month. Throughout the sampling period, 3524 individuals belonging to 39 species of Scarabaeinae were captured (Table 1). A total of 917 specimens were measured, usually 50 individuals per species, but for less abundant species all of the captured individuals were measured. Specimens were deposited in the Museu de Zoologia, Sao Paulo University and were identified by Fernando Z. Vaz-de-Mello.

### Geometric morphometric analyses

The Cartesian coordinates of anatomical landmarks are used in geometric morphometrics. These are specific locations on the organism's body, such as the points of convergence of structures, the apices of processes or their corresponding endpoints (Bookstein 1991). To study the difference in shape between two or more bodies (as described by landmark configurations), it is first necessary to plot the coordinates of the points as a figure in two or three-dimensional space on Cartesian axes. The resulting figure can then be thought of as a single point on a system of orthogonal axes, the number of which depends on the number of points that each figure has. This multidimensional space contains information about the shape, the size, position, and orientation of the body (Monteiro and Reis 1999).

To compare the shapes of two species' configurations, it is necessary to remove the information that does not pertain to shape, which means removing information about size, position, and orientation (Bookstein 1989). To eliminate the effect of size, the species configurations are scaled, making the centroid size of the figures equal to one. The centroid represents the species configuration midpoint or the centre of gravity of the figure, and its size is defined as the square root of the sum of the squares of the distances among each point of the species configuration and the centroid (Bookstein 1991). To eliminate the effects of position, the species configurations are translated to the same position in space, superimposing the centroid of one figure upon the centroid of another. The effect of orientation is eliminated by rotating the figures, following a criterion of optimization that minimizes the sum of the squares of the distances between homologous points, such that the rotation results in the minimum distance between the points of one over those of the other (Rohlf and Slice 1990).

### Data collection

**Figure 1.**
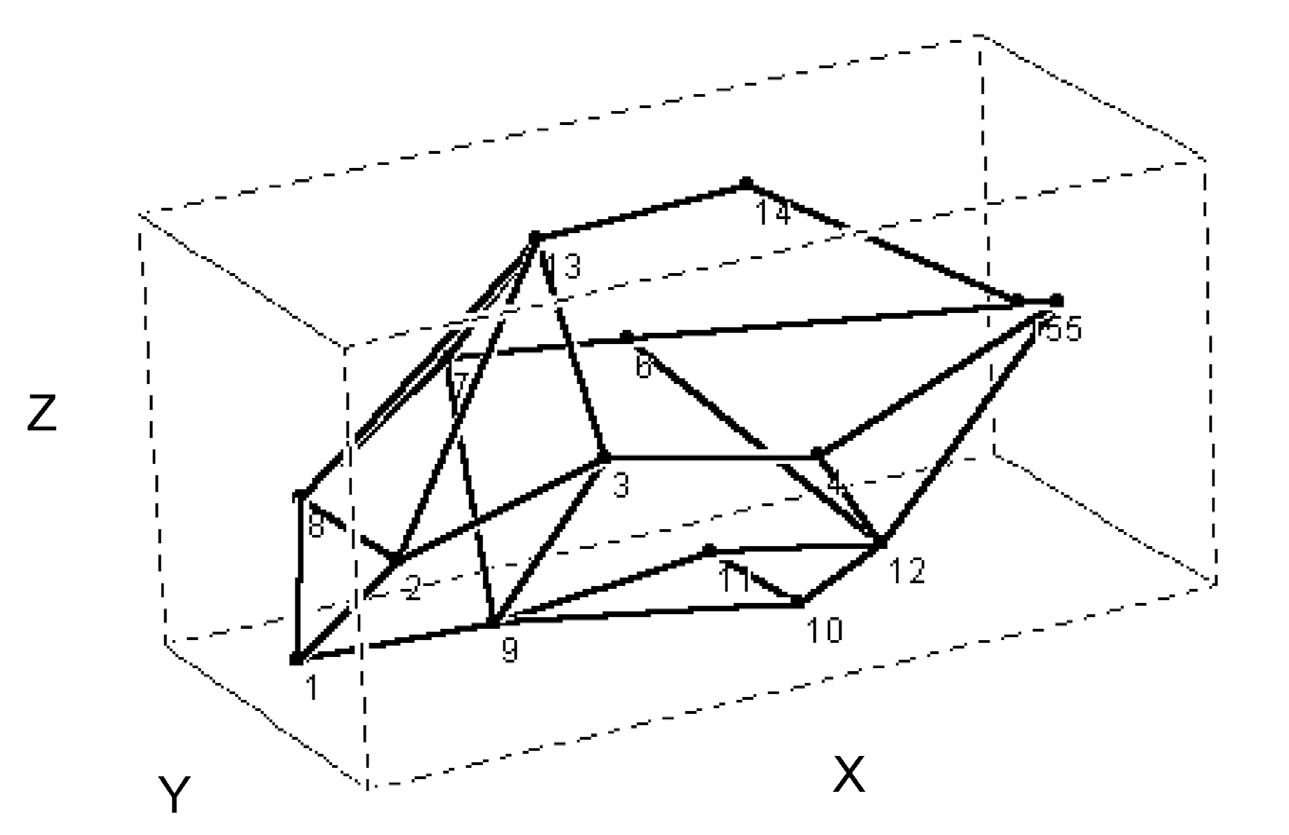
Average body shape for a synthetic Scarabaeinae beetle based on the configuration of 15 body landmarks in three-dimensional morphometric space. Landmark descriptions are given in Methods. The graphical representation of the body-plan can be observed from any angle, thereby facilitating the understanding of variability in body shape. High quality figures are available online.

Fifteen anatomical landmarks were selected on three-dimensional Cartesian coordinates to describe both the shape of the species and the size of the centroid. The following landmarks were chosen along homologous points in all the species: 1) anterior margin of the head; 2) eye position; 3) division between the pronotum and the elytra; 4) division between the thorax and the abdomen; 5) posterior margin of the abdomen; 9) point of insertion of the anterior legs; 10 and 11) points of insertion of the central legs; 12) point of insertion of the posterior legs; 13) anterior point of convergence between elytra; 14) central point (mid-line) of convergence between elytra, and 15) posterior margin (along mid-line) of the elytra. Points 6, 7, and 8 correspond to points 4, 3, and 2, respectively, for the other side of the body. The average shape of the body of dung beetles is shown in three-dimensional space in Figure 1. The coordinates of this synthetic body-plan were calculated from a global average based on all 39 species, and were used as reference points for describing the variation in shape among individual species. The data points were captured using a video camera and the program MorphoSys (Meacham and Duncan 1993), and a stereoscopic microscope was also used for individuals with a body length less than 8 mm.

After eliminating the information pertaining to size, position, and orientation, the residual information was used to characterize the body shape variables in a principal components analysis (PCA). Shape differences among species were visualized using icons representing the gradients along the major axes of variation. The similarities in shape among species were subjected to a hierarchical evaluation using the Procrustes distance in morphometric space in a cluster analysis with the Unweighted Pair-Group Average (UPGMA) method.

The size of each species was measured from the size of the centroid (average of all sample individuals) — a measurement that is considered to be a geometrically robust representation of the size of insects for which morphology is often complex (Bookstein 1982, 1991). However, to allow for the comparison of the results of this study with those of other studies, body length was measured as the distance from the anterior margin of the head to the posterior edge of the abdomen (the distance between points 1 and 5 of the landmark configurations).

The relationship between body size and body shape was analyzed separately for diurnal and nocturnal species. To this end, first the diel activity was noted for each species according to Hernández (2002), who identified the diurnal and nocturnal species of the Serra do Japi. Then, to visualize the relationship between body size and body shape for each guild, the size of each species (x axis) was graphed in relation to the first PCA axis obtained in the previous analysis. This allowed direct comparison of differences in body size and shape among the species which supposedly compete more intensely at the same time for the same resource.

## Results

**Figure 2.**
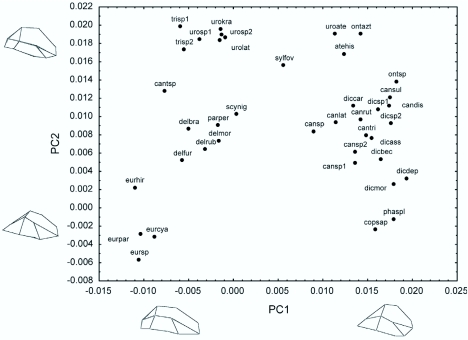
Principal Components Analysis (PCA) illustrating differences in body shape for 39 species of Scarabaeinae. A visual representation of the gradients in body shape along the principal axes is provided by two-dimensional sketch diagrams. High quality figures are available online.

The principal components analysis revealed a gradient which describes the greatest amount of variation in body shape among species. The first component explains 38.5% of the variation in shape, and represents a gradient between species which are elongated and flattened with a narrow abdomen (negative scores) and those with a rounded or convex body shape (positive scores). The second component explains 17.8% of the remaining variation, and represents a gradient between species with an abdomen that is proportionally larger than the elytra (negative scores) versus species with proportionally large elytra but constricted abdomens (positive scores, Figure 2).

The species with the lowest scores on the first and second components belong to the genus *Eurysternus* and these have the most elongated shape and flattest bodies with a narrow abdomen, but their abdomen is proportionally larger than their elytra. The species of the genus *Deltochilum* are more gently elongated, as are those of *Canthonella* sp., *Paracanthon pereirai*, and *Scybalocanthon nigriceps* (Figure 2, see Table 1 for codes assigned to species). The species of *Uroxys* (except *U. aterrima*) and *Trichillum* are also more gently elongated, but as they have the highest scores on the second component, they have proportionally large elytra and a constricted abdomen. *Coprophanaeus saphirinus* and *Phanaeus splendidulus* have high scores on the first component, but low on the second component, with rounded or convex body shapes and a proportionally larger abdomen than elytra. Species from the genera *Dichotomius, Canthidium, Canthon*, and *Onthophagus* have high scores on the first component and increasing scores along the second component, so they tend to have rounded body shapes and large elytra with a constricted abdomen. *Ateuchus histrio, Ontherus azteca*, and *Uroxys aterrima* have high scores on the first and second components, with rounded or convex body shapes and proportionally large elytra and a constricted abdomen.

**Figure 3.**
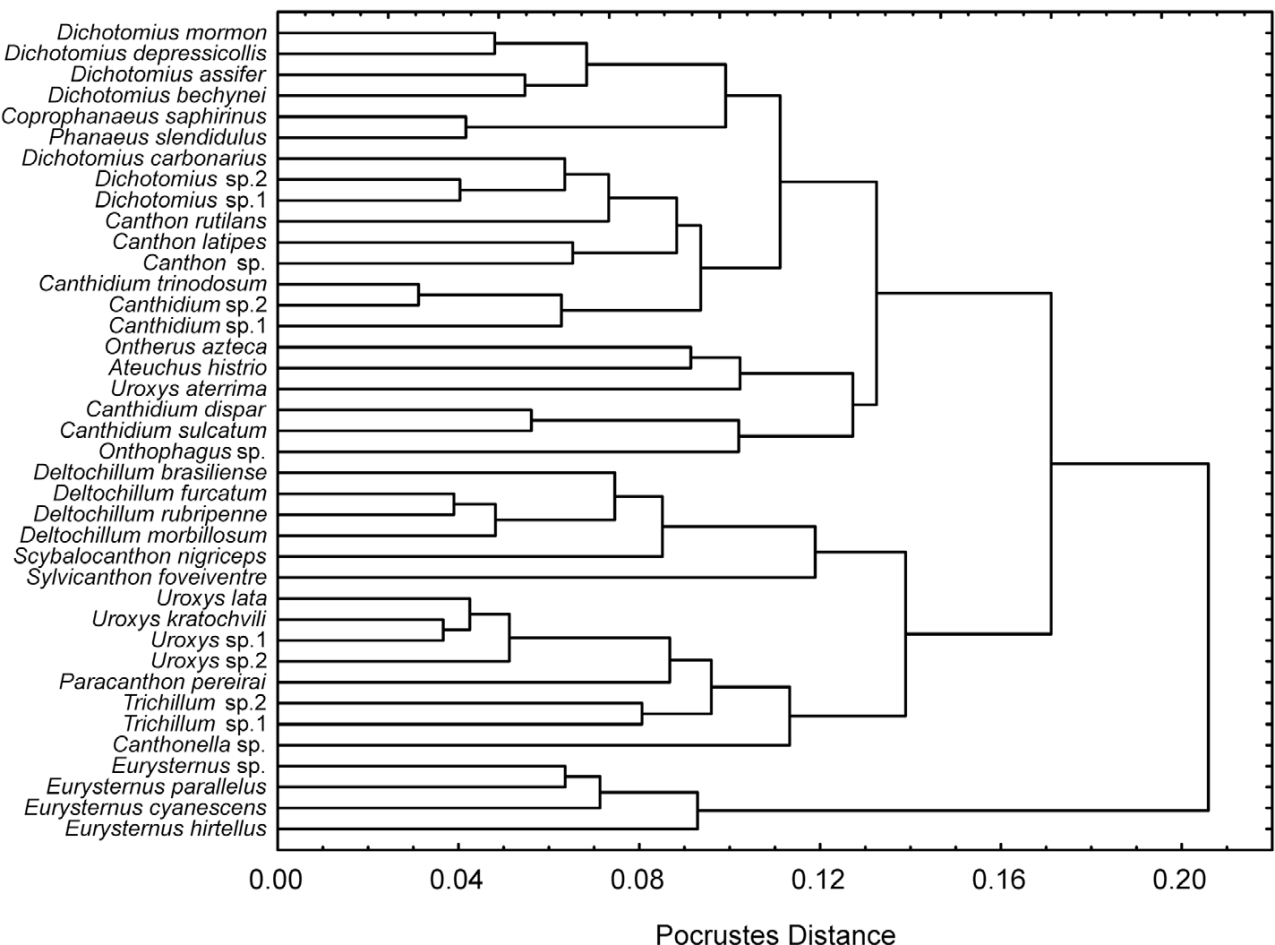
UPGMA cluster analysis based on a Procrustes distance matrix defining differences in body shape for 39 sympatric Scarabaeinae species from Serra do Japi, Brazil. High quality figures are available online.

As a complement to the PCA, the cluster analysis allowed a simultaneous evaluation of all 15 morphological landmarks based on the underlying similarity matrix for different species (Figure 3). There was a tendency to group species according to their taxonomy, with closely related species clustering tightly together. This pattern was most evident for *Eurysternus* species which, due to their unique shape, were grouped together in an isolated cluster separate from all other species. However, in spite of this general pattern, the location of some species does not correspond to their taxonomy. Some species from different tribes have similar shapes, while others from the same tribe have contrasting shapes. Two large, but distinct taxonomic groups were identified as having similar body shapes (Figure 3). The first included all the species of tribe Canthonini (except *Canthon*) together with species of the genera *Uroxys* and *Trichillum* (tribe Ateuchini, except *U*. *aterrima*). The second group included all other species of Ateuchini, together with the Coprini (*Dichotomius, Ontherus*) and Phanaeini (*Coprophanaeus, Phanaeus*), as well as all the species of the genus *Canthon* (tribe Canthonini) (Figure 3). These results concur with those of the PCA.

Measurements of body size (centroid size) and body length are given in Table 1. There was a positive and highly significant correlation (r^2^ = 0.998, p < 0.001) between body length and the size of the centroid, represented by the linear equation: centroid size = 0.031 + 1.247 × length.

**Figure 4.**
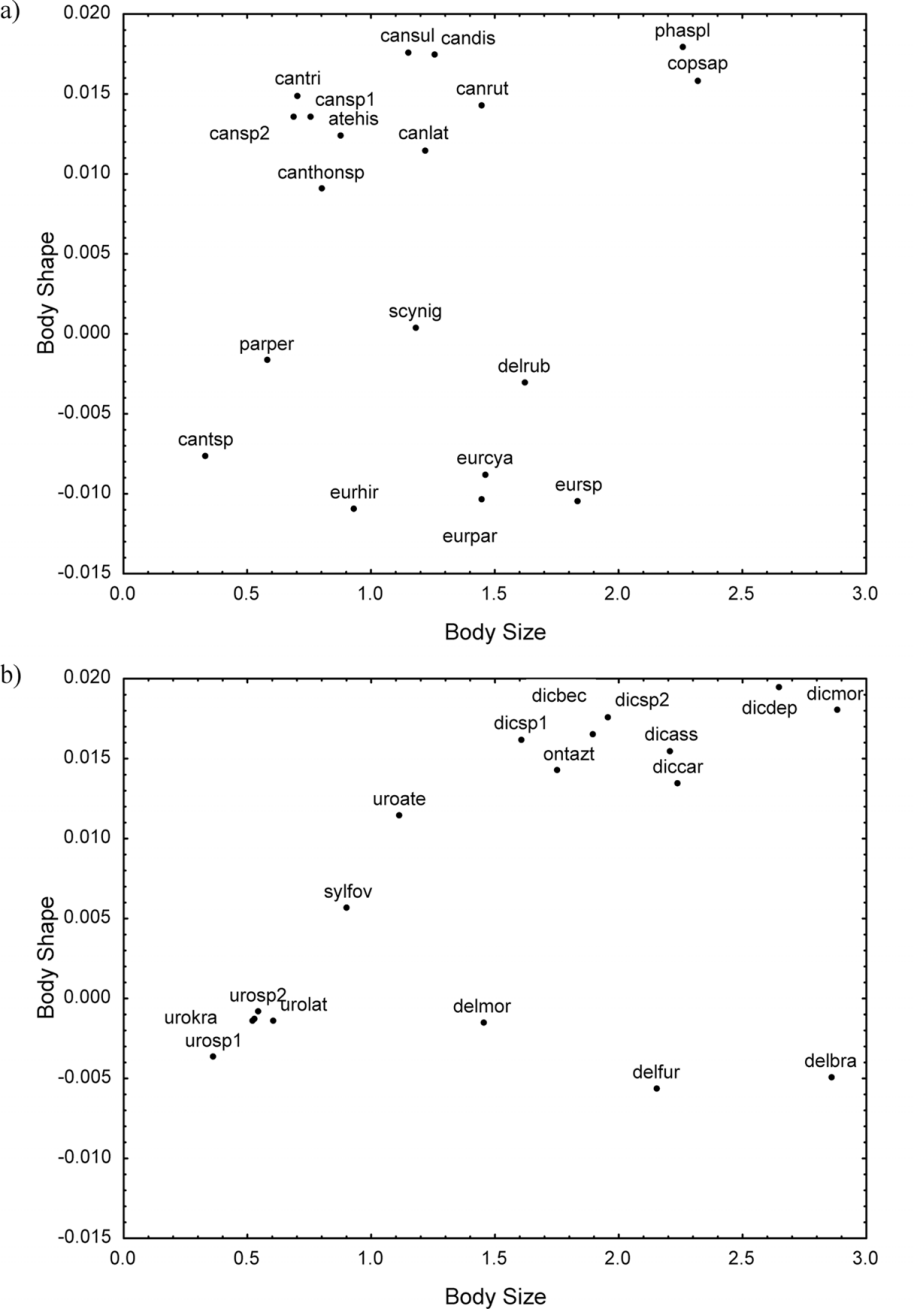
Relationship between body size (i.e. the size of the centroid in cm) and body shape (first principal component in the PCA) in (a) the diurnal and (b) nocturnal dung beetle species in Serra do Japi, Brazil. High quality figures are available online.

The relationships between body size and body shape (defined by the first principal component) show that the 19 diurnal species were divided into two major groups as defined by differences in body shape, though there is notable variability in size within each group (Figure 4a). In addition, each species is located at a unique coordinate as defined by its size and morphology. A similar pattern was found for the 17 nocturnal species, but the range of body size was broader than that of the diurnal species.

**Table 1.**
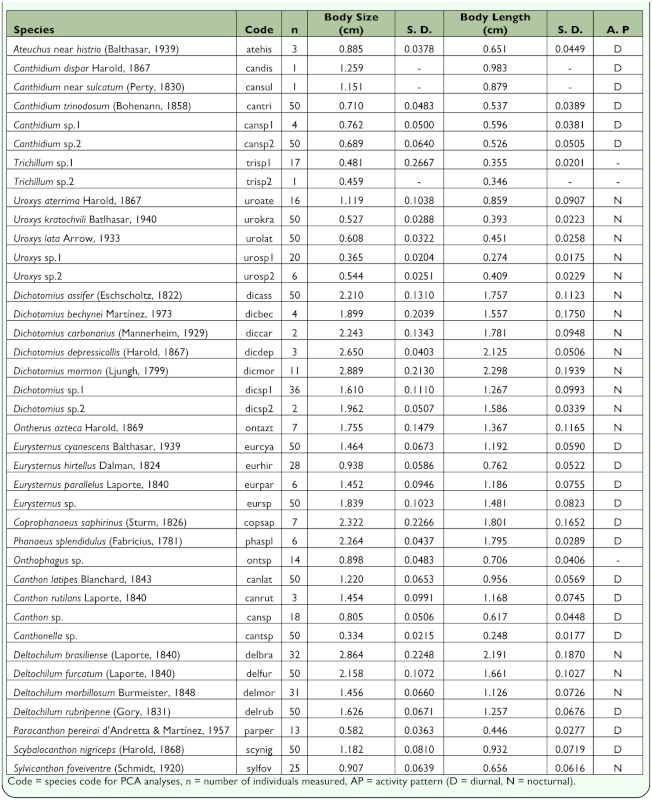
Species of Scarabaeinae captured in the Atlantic Forest at the Serra do Japi, Brazil. Body size is defined by the size of the centroid.

Additionally, nocturnal species have more convex and bigger bodies than diurnal species (Figure 4b). The species of *Canthidium* (diurnal) and *Uroxys* (nocturnal) were respectively grouped very closely together indicating very little variability in shape or size within each genus.

## Discussion

The assemblage of Scarabaeinae beetles in the Serra do Japi is characterized by high levels of diversity and abundance (Hernández and Vazde-Mello 2009) and, as results show, high diversity in body size and morphology. This structure is typical of other tropical dung beetle assemblages (Halffter and Matthews 1966; Hanski 1991; Gill 1991; Halffter *et al*. 1992; Davis *et al*. 2001; Endres *et al*. 2007; Gardner *et al*. 2008; Navarrete and Halffter 2008), and competition is presumably involved in their structuring (Hanski and Cambefort 1991). However, the few studies conducted with dung beetles that have experimentally analyzed competition under field and laboratory conditions (Giller and Doube 1986; Horgan and Fuentes 2005; Slade *et al*. 2008), do not explicitly include the morphology of the species in their analysis. Based on the results presented here, the incorporation of this approach, together with species size, allows better understanding of the role of species morphology in the intraand interspecific interactions that occur in dung beetle assemblages.

Within a single community, species from the same taxonomic group that have a similar body size often exploit similar resources and are therefore likely to compete more strongly with each other than with species which are less similar (Warren and Lawton 1987). In Serra do Japi, the Scarabaeinae belonging to the same genera and sharing a similar body shape clustered together in both the principal components and cluster analyses. This was particularly evident for the species of *Eurysternus, Canthidium, Uroxys*,*Dichotomius*, the *Phanaeus*, and the roller species belonging to *Canthon* and *Deltochilum*, suggesting that competition should be strong among these taxonomic groups. However, although such similarities in body shape suggest that the species of a genus employ similar life history strategies to exploit common resources, the fact that there are differences in body size also suggest asymmetric competition between species of the same clade. In contrast to this general rule there were clusters of species that were not related at the genus level, but that shared similar body shape. An example of this is the group formed by *Ontherus azteca, Atheuchus histrio*, and *Uroxys aterrima*. However, this does not necessarily imply intense competition between these species because they differ in body size and in their foraging strategies.

There are two key limiting factors in relation to the size of species within a given community. Small individuals are limited by their physical capacity to acquire resources, but they are efficient at converting food into reproductive output. In contrast, large individuals are effective at securing resources, but are much less effective at exploiting them for reproduction. These two processes often result in the evolution of an optimal size that characterizes the majority of species (Brown *et al*. 1993). There is considerable variation in size in the assemblage of dung beetles in the Serra do Japi (as defined by the centroid size), ranging from the smallest species (e.g., *Canthonella* sp.) with a size of 3 mm to the largest with an average body size of 3 cm (*Dichotomius mormon*). However, the centroid size of more than half (21) of the coexisting 39 species was between 0.5 and 1.5 cm, suggesting that this is the optimal size interval for the assemblage of dung beetles in the Serra do Japi. These results also suggest that the structure of the dung beetle assemblage from the Serra do Japi mainly reflects a variance-covariance dynamic; one in which many species do not use up their patchily available resources quickly, but rather compete for them over a prolonged period of time (see Hanski 1991).

The relationship found between body size and body shape in this study revealed that some species are very similar in body shape but have marked differences in body size, confirming predictions for both diurnal and nocturnal species. This fact, together with the etoecological differences among the cooccurring species of dung beetles from the Serra do Japi may facilitate their coexistence, and this is evident within and between diurnal and nocturnal species. In conclusion, the geometric morphometric analysis suggests that body size and body shape are important factors that should be incorporated into studies on the structure of dung beetle assemblages. Alternative life history strategies for exploiting common resources depend to a large extent upon differences in body size and body shape among species in the same functional group and generate asymmetric competitive interactions. Linking the behavior, body size, morphology, and life history of dung beetles, as well as phylogenetic and empirical studies, will help achieve a better understanding of how dung beetle assemblages are structured in natural and disturbed ecosystems.

## References

[bibr01] Adams DC, Funk DJ (1997). Morphometric inferences on sibling species and sexual dimorphism in *Neochlamisus bebbianae* leaf beetles: multivariate applications of the thinplate spline.. *Systematic Biology*.

[bibr02] Begon M, Harper JL, Townsend CR (1996). Ecology, individuals, populations and communities..

[bibr03] Blackburn TM, Gaston KJ (1997). A critical assessment of the form of the interspecific relationship between abundance and body size in animals.. *Journal of Animal Ecology*.

[bibr04] Bookstein FL (1982). Foundations of morphometrics.. *Annual Review of Ecology and Systematics*.

[bibr05] Bookstein FL (1989). “Size and shape”: a comment on semantics.. *Systematic Zoology*.

[bibr06] Bookstein FL (1991). *Morphometric Tools for Landmark Data: Geometry and Biology*..

[bibr07] Brown JH, Marquet PA, Taper ML (1993). Evolution of body size: Consequences of an energetic definition of fitness.. *American Naturalist*.

[bibr08] Calder WA (1984). *Size, Function, and Life History*..

[bibr09] Camberfort Y (1985). Les Coléoptéres Scarabaeidae du Parc National de Taï (Côte d'Ivoire).. *Revue Francaise d'Entomologie*. (N.
S).

[bibr10] Cambefort Y (1986). Rôle des coléoptéres Scarabaeidae dans l'enfouissement des excréments en savane guinéenne de Côte d'Ivoire.. *Acta Oecologica, Oecologia Generalis*.

[bibr11] Davis ALV (1996). Community organization of dung beetles (Coleoptera: Scarabaeidae): differences in body size and functional group structure between habitats.. *African Journal of Ecology*.

[bibr12] Endres AA, Creão-Duarte AJ, Hernández MIM (2007). Diversidade de Scarabaeidae *s. str*. (Coleoptera) da Reserva Biológica Guaribas, Mamanguape, Paraíba, Brasil: uma comparação entre Mata Atlântica e Tabuleiro Nordestino.. *Revista Brasileira de Entomologia*.

[bibr13] Feer F, Pincebourde S (2005). Diel flight activity and ecological segregation within an assemblage of tropical forest dung and carrion beetles.. *Journal of Tropical Ecology*.

[bibr14] Gardner TA, Hernández MIM, Barlow J, Peres CA (2008). Understanding the biodiversity consequences of habitat change: the value of secondary and plantation forests for neotropical dung beetles.. *Journal of Applied Ecology*.

[bibr15] Gause GF (1934). The Struggle for Existence.. http://www.ggause.com/Contgau.htm.

[bibr16] Gill BD, Hanski I, Cambefort Y (1991). Dung Beetles in Tropical American Forest.. *Dung Beetle Ecology*.

[bibr17] Giller PS, Doube BM (1989). Experimental analysis of inter- and intraspecific competition in dung beetle communities.. *Journal of Animal Ecology*.

[bibr18] Halffter G (1991). Historical and ecological factors determining the geographical distribution of beetles (Coleoptera: Scarabaeidae: Scarabaeinae).. *Folia Entomológica Mexicana*.

[bibr19] Halffter G, Matthews EG (1966). The natural history of dung beetles of the subfamily Scarabaeinae (Coleoptera, Scarabaeidae).. *Folia Entomológica Mexicana*.

[bibr20] Halffter G, Edmonds WD (1982). *The Nesting Behavior of Dung Beetles* (*Scarabaeinae*): *An Ecological and Evolutive Approach*.. Instituto de Ecología..

[bibr21] Halffter G, Halffter V, Huerta C (1980). Mating and nesting behaviour of *Eurysternus* (Coleoptera: Scarabaeinae).. *Quaestiones Entomologicae*.

[bibr22] Halffter G, Favila ME, Halffter V (1992). A comparative study of the structure of the scarab guild in Mexican tropical rain forest and derived ecosystems.. *Folia Entomológica Mexicana*.

[bibr23] Hanski I, Hanski I, Cambefort Y (1991). The Dung Insect Community.. *Dung Beetle Ecology*.

[bibr24] Hanski I, Koskela H (1977). Niche relations among dung-inhabiting beetles.. *Oecologia*.

[bibr25] Hanski I, Cambefort Y, Hanski I, Cambefort Y (1991). Resource Partitioning.. *Dung Beetle Ecology*.

[bibr26] Hernández MIM (2002). The night and day of dung beetles (Coleoptera, Scarabaeidae) in the Serra do Japi, Brazil: elytra colour related to daily activity.. *Revista Brasileira de Entomologia*.

[bibr27] Hernández MIM, Vaz-de-Mello FZ (2009). Seasonal and spatial variation of coprophagous Scarabaeidae *s. str*. (Coleoptera) species richness in areas of Atlantic forest of the state of São Paulo, Brazil.. *Revista Brasileira de Entomologia*.

[bibr28] Horgan FG, Fuentes RC (2005). Asymmetrical competition between Neotropical dung beetles and its consequences for assemblage structure.. *Ecological Entomology*.

[bibr29] Hutchinson GE, MacArthur RH (1959). A theoretical ecological model of size distributions among species of animals.. *The American Naturalist*.

[bibr30] Juliano SA, Lawton JH (1990). The relationship between competition and morphology II. Experiments on co-occurring dysticid beetles.. *Journal of Animal Ecology*.

[bibr31] Lawton JH, Price PW, Lewinsohn TM, Fernandes GW, Benson WW (1991). Species richness, population abundances, and body sizes in insect communities: tropical versus temperate comparisons.. *Plant-Animal Interactions*.

[bibr32] Meacham C, Duncan T (1993). *MorphoSys. Automate Morphometric System*..

[bibr33] Monteiro LR, Reis SF (1999). *Principios de Morfometria Geométrica*..

[bibr34] Morse DR, Stork NE, Lawton JH (1988). Species number, species abundance and body length relationship of arboreal beetles in Bornean lowland rain forest trees.. *Ecological Entomology*.

[bibr35] Palestrini E, Barbero E, Rolando A (1998). Intra- and interspecific aggregation among dung beetles (Coleoptera: Scarabaeoidea) in an Alpine pasture.. *Journal of Zoology*.

[bibr36] Peck SB, Forsyth A (1982). Composition, structure and competitive behaviour in a guild of Ecuadorian rain forest dung beetles (Coleoptera: Scarabaeidae).. *Canadian Journal of Zoology*.

[bibr37] Peters RH (1983). *The Ecological Implications of Body Size*..

[bibr38] Pretorius E, Scholtz CH (2001). Geometric morphometrics and the analysis of higher taxa: a case study based on the metendosternite of the Scarabaeoidea (Coleoptera).. *Biological Journal of the Linnean Society*.

[bibr39] Pretorius E, Philips K, Scholtz CH (2003). Geometric morphometries, the metendosternite and its uses in phylogenetics of the Scarabaeinae (Coleoptera).. *Elytron*.

[bibr40] Rohlf FJ (1993). *NTSYS-pc*, version 1.80. Exeter Software..

[bibr41] Rohlf FJ (1999). Shape statistics: Procrustes superimposition and tangent spaces.. *Journal of Classification*.

[bibr42] Rohlf FJ, Slice DE (1990). Extensions of the Procrustes method for the optimal superimposition of landmarks.. *Systematic Zoology*.

[bibr43] Rohlf FJ, Marcus LF (1993). A revolution in morphometrics.. *Trends in Ecology and Evolution*.

[bibr44] Slice DE (1994). GRF-ND. *Generalized rotational fitting of n-dimensional landmark data*..

[bibr45] Schmidt-Nielsen K (1984). *Scaling, why is animal size so important?*.

[bibr46] Spector S (2006). Scarabaeinae dung beetles (Coleoptera: Scarabaeidae: Scarabaeinae): an invertebrate focal taxon for biodiversity research and conservation.. *The Coleopterists Bulletin*.

[bibr47] Tilman D, May RM, McLean AR (2007). Interspecific competition and multispecies coexistence.. *Theoretical ecology principles and applications*.

[bibr48] Vandermeer J, Evans MA, Foster P, Hook T, Wund M (2002). Increased competition may promote species coexistence.. *Proceedings of the National Academy of Science*.

[bibr49] Vaz-de-Mello FZ, Martín-Piera F, Morrone JJ, Melic A (2000). Estado atual de conhecimento dos Scarabaeidae *s. str*.
(Coleoptera: Scarabaeoidea) do Brasil.. *Hacia un Proyecto CYTED para el Inventario y Estimación de la Diversidad Entomológica en Iberoamérica: PrIBES-2000*.

[bibr50] Warren PH, Lawton SH (1987). Invertebrate predator-prey body size relationship: an explanation for upper triangular food webs and patterns in food web structure?. *Oecologia*.

